# C_60_ Fullerene Reduces the Level of Liver Damage in Chronic Alcohol Intoxication of Rats

**DOI:** 10.3390/molecules29132951

**Published:** 2024-06-21

**Authors:** Olexandr Motuziuk, Dmytro Nozdrenko, Svitlana Prylutska, Igor Vareniuk, Vsevolod Cherepanov, Kateryna Bogutska, Sergii Rudenko, Yuriy Prylutskyy, Jacek Piosik, Uwe Ritter

**Affiliations:** 1 ESC “Institute of Biology and Medicine”, Taras Shevchenko National University of Kyiv, Str. Volodymyrska 64/13, 01601 Kyiv, Ukraine; motuziuk.oleksandr@eenu.edu.ua (O.M.); dmytro.nozdrenko@knu.ua (D.N.); vareniukim@knu.ua (I.V.); bogutska_ki@knu.ua (K.B.); rudenkosrg@gmail.com (S.R.); prylut@ukr.net (Y.P.); 2 Faculty of Biology and Forestry, Lesya Ukrainka Volyn National University, Av. Voli 13, 43025 Lutsk, Ukraine; 3 Faculty of Plant Protection, Biotechnology and Ecology, National University of Life and Environmental Science of Ukraine, Str. Heroiv Oborony 13, 03041 Kyiv, Ukraine; psvit_1977@ukr.net; 4 Institute of Physics, NAS of Ukraine, Av. Nauki 46, 03028 Kyiv, Ukraine; vvch2000@ukr.net; 5 Intercollegiate Faculty of Biotechnology, University of Gdansk, Str. Abrahama 58, 80-307 Gdańsk, Poland; jacek.piosik@ug.edu.pl; 6 Institute of Chemistry and Biotechnology, Technical University of Ilmenau, Str. Weimarer 23, 98693 Ilmenau, Germany

**Keywords:** C_60_ fullerene, liver, alcohol intoxication, biochemical blood parameters, histopathological analysis, atomic force microscopy

## Abstract

The liver is the main organ responsible for the metabolism of ethanol, which suffers significantly as a result of tissue damage due to oxidative stress. It is known that C_60_ fullerenes are able to efficiently capture and inactivate reactive oxygen species in in vivo and in vitro systems. Therefore, the purpose of this study is to determine whether water-soluble C_60_ fullerene reduces the level of pathological process development in the liver of rats induced by chronic alcohol intoxication for 3, 6, and 9 months, depending on the daily dose (oral administration; 0.5, 1, and 2 mg/kg) of C_60_ fullerene throughout the experiment. In this context, the morphology of the C_60_ fullerene nanoparticles in aqueous solution was studied using atomic force microscopy. Such biochemical parameters of experimental animal blood as ALT (alanine aminotransferase), AST (aspartate aminotransferase), GGT (gamma-glutamyl transferase) and ALP (alkaline phosphatase) enzyme activities, CDT (carbohydrate-deficient transferrin) level, values of pro-antioxidant balance indicators (concentrations of H_2_O_2_ (hydrogen peroxide) and GSH (reduced glutathione), activities of CAT (catalase), SOD (superoxide dismutase) and GP_x_ (selenium-dependent glutathione peroxidase)), and pathohistological and morphometric features of liver damage were analyzed. The most significant positive change in the studied biochemical parameters (up to 29 ± 2% relative to the control), as markers of liver damage, was recorded at the combined administration of alcohol (40% ethanol in drinking water) and water-soluble C_60_ fullerenes in the optimal dose of 1 mg/kg, which was confirmed by small histopathological changes in the liver of rats. The obtained results prove the prospective use of C_60_ fullerenes as powerful antioxidants for the mitigation of pathological conditions of the liver arising under prolonged alcohol intoxication.

## 1. Introduction

The liver is the main organ responsible for the metabolism of ethanol, which suffers largely as a result of tissue damage due to oxidative stress. Alcohol-related liver diseases include fatty liver or steatosis, steatohepatitis, alcoholic hepatitis, and cirrhosis [1]. Alcohol metabolism also occurs in other organs, for example heart, brain, kidneys, pancreas, skeletal muscle, lungs, red blood cells, lymphocytes, etc. However, most studies on chronic alcoholism point to the leading role of the liver in increasing oxidative stress and lipid peroxidation (LPO) [1]. Liver microsomes can oxidize ethanol in an NADH-dependent pathway catalyzed by iron [2,3]. The resulting acetaldehyde affects the immune system by altering the production of cytokines, antioxidant enzymes, and substances, especially mitochondrial and cytosolic glutathione. These compounds can cause toxic effects, as the proteins they bind to lose their functions and provoke an immune response [4].

Chronic alcohol consumption causes hypoxia in the pericentral liver, where additional oxygen is required for alcohol metabolism [5]. Hypoxia causes additional production of reactive oxygen species (ROSs) and LPO through the activity of xanthine oxylase in the liver [6]. In addition, a high alcohol concentration facilitates the absorption of endotoxins by altering intestinal permeability, modifying the intestinal microflora, and directly damaging the cells lining the inner gut [7]. Endotoxins physically attach to the surface of Kupffer cells via the CD14 receptor and Toll-like receptor 4 (TLR4) [8]. The results of study [9] indicate that the oxidative stress associated with chronic alcohol consumption can be explained by the activation of Kupffer cells by endotoxins.

The toxic effect of ethanol is in direct correlation with the concentration of acetaldehyde and acetate in the blood. During ethanol oxidation, there is an increased consumption of coenzyme NAD^+^, and an increase in the NADH/NAD^+^ ratio, that plays an important role in the formation of fatty liver dystrophy. Acetaldehyde has a hepatotoxic effect, which is manifested due to the intensification of LPO processes, and the formation of compounds with other proteins and enzymes, which leads to the disruption of the function of phospholipid cell membranes. The complex of acetaldehyde compounds with proteins, including tubulin, causes changes in the structure of hepatocyte microtubules, forming the so-called alcoholic hyaline, and contributes to the disruption of intracellular transport, protein and water retention, and the development of balloon dystrophy of hepatocytes [10].

The current conventional diagnosis of alcoholic liver disease is based on a history of alcohol consumption and laboratory results of blood biochemical diagnostic parameters such as ALT (alanine aminotransferase), AST (aspartate aminotransferase), and GGT (gamma-glutamyl transferase) [11,12]. However, it is not possible to accurately determine the extent of liver damage while analyzing these biomarkers.

It is known that biocompatible and bioavailable C_60_ fullerene nanoparticles can efficiently capture and inactivate ROSs in in vivo and in vitro systems [13,14]. Recently it was demonstrated that applying water-soluble C_60_ fullerenes during chronic alcoholization of rats reduces the level of fluctuations of force muscle response (the so-called “tremor”) [15] and protects muscle tissue from damage caused by oxidative stress [16]. 

Thus, the purpose of this study was to determine whether water-soluble C_60_ fullerene reduces the level of pathological processes in the liver of rats caused by chronic alcohol intoxication, depending on the oral administration dose (“dose–effect”) together with alcohol throughout the experiment. In this context, the basic biochemical indices of experimental animal blood, as markers of liver damage, as well as the pathohistological and morphometric features of liver damage, were analyzed.

## 2. Results and Discussion 

### 2.1. AFM Study 

**Figure 1:**
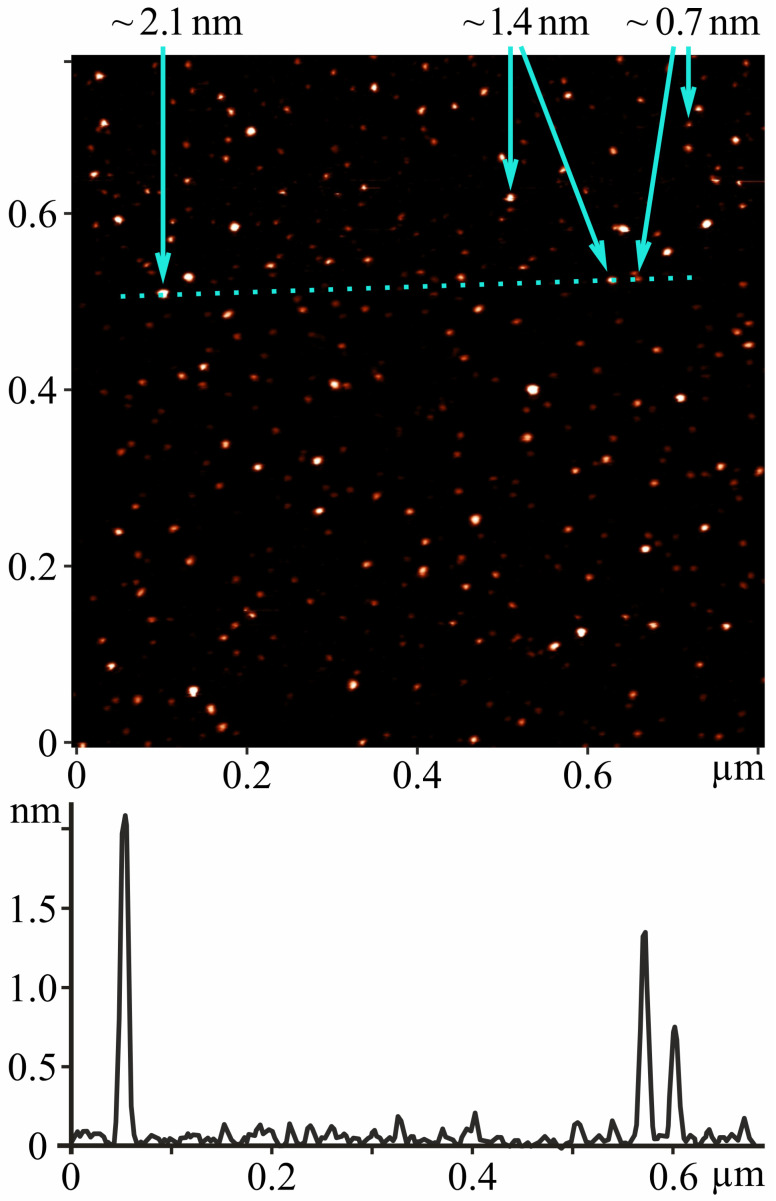
(**Top**): AFM image of single C_60_ molecules and their nanoaggregates deposited from C_60_FAS (0.15 mg/mL) on a mica substrate. The arrows indicate the height of the nanoobjects (height ~0.7 nm corresponds to the diameter of the C_60_ molecule). (**Bottom**): *Z*-profile of the image along the marked dotted line.

Atomic force microscopy (AFM) measurements showed that the C_60_ fullerene layer consisted of spatially separated randomly located point objects (Figure 1). The height of most of them did not exceed 2.1 nm. As can be seen in the *Z*-profile, the widths of the objects were in the range of 1.0–1.5 nm, which is close to the typical tip radius of the AFM probe used in the experiments. This allowed us to identify objects with a height of ~0.7 nm as individual C_60_ molecules, and higher objects as aggregates of molecules with a shape close to spherical. The size of most aggregates did not exceed three molecular diameters. Thus, according to the AFM data, C_60_ fullerene was in a molecular or low-aggregated state in the characterized solution. The isolated arrangement of C_60_ molecules (nanoclusters) can be explained by the existence of electrostatic repulsion forces between them. They exhibited a high negative surface charge (zeta potential value was −25.3 mV at room temperature [17]), indicating a low tendency for aggregation in aqueous solution (i.e., a high solute stabilization). 

Thus, the detected nanoformation of the C_60_ molecules in an aqueous solution (0.15 mg/mL) indicates their suitability for in vivo studies.

### 2.2. BAC Value 

The blood alcohol concentration (BAC) in animals after chronic ethanol consumption ranged from 142 ± 4 mg/dL (3 months of alcohol consumption) to 257 ± 5 mg/dL (9 months of alcohol consumption). These results agree well with the data [18]. C_60_ fullerene aqueous solution (C_60_FAS) administration at a dose range of 0.5, 1, and 2 mg/kg together with ethanol did not lead to a reliable change in BAC values. 

### 2.3. Biochemical Analysis

During chronic alcohol consumption, increased accumulation of LPO products, ROSs, glycosylated proteins, and carbohydrates leads to the formation of oxidative stress and may be the cause of cytolysis of hepatocytes, the main markers of which are the liver enzymes ALT, AST, and ALP (alkaline phosphatase) [19]. 

ALT is an enzyme whose highest amount is produced by the liver, heart muscle, pancreas, kidney, and muscle cells, so its increase in blood indicates damage to these organs [20].

**Figure 2:**
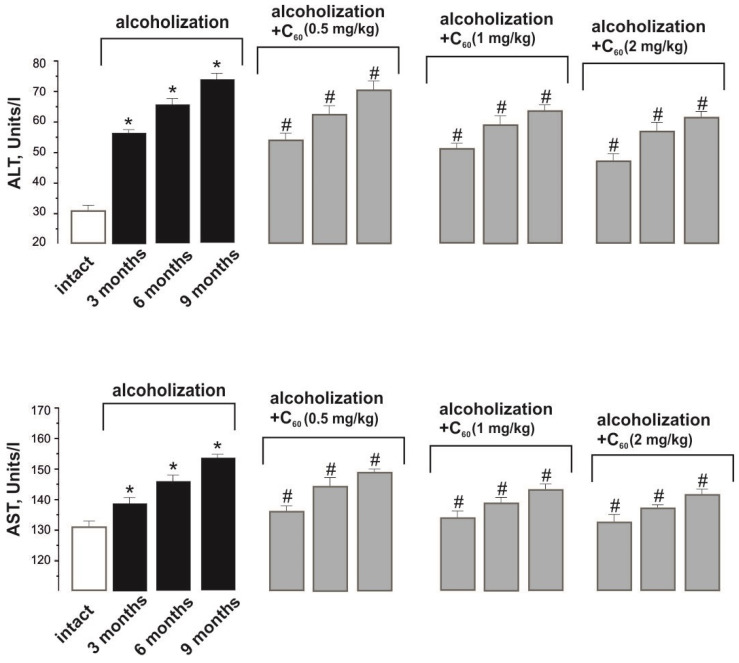
Activities of AST and ALT in the blood of alcoholized animals: intact—rats receiving 100% drinking water; alcoholization—alcoholized rats (control); alcoholization+C_60_ (0.5, 1 and 2 mg/kg)—rats receiving a mixture of ethanol and C_60_FAS at doses of 0.5, 1, and 2 mg/kg, respectively, throughout alcoholization; 3, 6, and 9 months—alcoholization of 3, 6, and 9 months, respectively. * *p* < 0.05 relative to the intact group; # *p* < 0.05 relative to the alcoholization group.

ALT activity increased from 31 ± 1 Units/L in the norm (intact group) to 56 ± 2, 67 ± 3, and 74 ± 3 Units/L during alcoholization lasting 3, 6, and 9 months (control), respectively (Figure 2). The co-administration of ethanol and C_60_FAS (dose 1 mg/kg) decreased its activity by 11 ± 1%, 16 ± 1%, and 18 ± 1% during alcoholization of 3, 6, and 9 months, respectively, relative to the control. This index did not significantly change with an increase in the dose of C_60_FAS to 2 mg/kg, and reducing the dose of C_60_FAS to 0.5 mg/kg decreased its positive effect by 11 ± 1% relative to the group “alcoholization+C_60_ (1 mg/kg)”.

AST is an enzyme, the largest amount of which is produced by liver and heart cells in pathology. Normally only a small part of this enzyme is present in the blood [20]. 

The increase in AST activity from 132 ± 1 Units/L in the norm (intact group) to 138 ± 6, 142 ± 7, and 154 ± 6 Units/L during alcoholization lasting 3, 6, and 9 months (control), respectively, is a consequence of progressive destruction of hepatocytes (Figure 2). The co-administration of ethanol and C_60_FAS (dose 1 mg/kg) decreased its activity by 10 ± 1% during the experiment relative to the control. This index did not significantly change with an increase in the dose of C_60_FAS to 2 mg/kg, and reducing the dose of C_60_FAS to 0.5 mg/kg decreased its positive effect by 10 ± 1% relative to the group “alcoholization+C_60_ (1 mg/kg)”.

In clinical practice, the ratio of AST to ALT activity in the blood (de Ritis ratio = AST/ALT) is used to diagnose liver diseases [20]. As mentioned above, these enzymes have organ specificity; in particular, in case of liver damage due to alcohol intoxication, increased activity of ALT in blood is detected.

The decrease in the de Ritis ratio from 0.97 ± 0.05 in the norm (intact group) to 0.80 ± 0.07, 0.68 ± 0.07, and 0.78 ± 0.07 at 3, 6, and 9 months of alcoholization indicates progressive disturbance of hepatocyte functioning, which is in good agreement with the data [20]: at the initial stage of alcohol intoxication, the de Ritis ratio was equal to 0.6. The co-administration of ethanol and C_60_FAS (dose 1 mg/kg) increased the de Ritis ratio by 14 ± 1% during the experiment relative to the control (ALT activity decreases more strongly than AST activity). 

GGT is an enzyme, the largest amount of which is accumulated in the liver, kidney, and pancreatic cells. It takes part in the metabolism of amino acids and synthesizing protein molecules. Unlike other liver enzymes, GGT production is “triggered” by alcohol, so its activity may be increased in abusers even in the absence of liver disease. This enzyme is more sensitive to disturbances in liver cells than ALT and AST [21].

**Figure 3:**
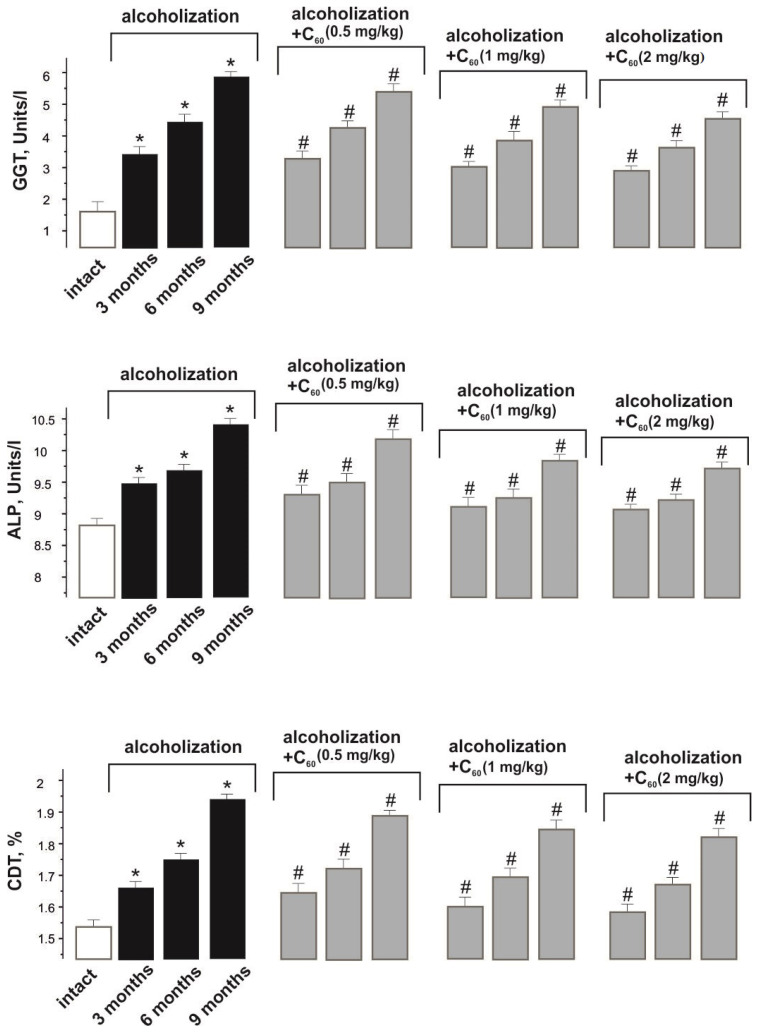
GGT and ALP activities and CDT level in the blood of alcoholized animals: intact—rats receiving 100% drinking water; alcoholization—alcoholized rats (control); alcoholization+C_60_ (0.5, 1, and 2 mg/kg)—rats receiving a mixture of ethanol and C_60_FAS at doses of 0.5, 1, and 2 mg/kg, respectively, throughout alcoholization; 3, 6, and 9 months—alcoholization of 3, 6, and 9 months, respectively. * *p* < 0.05 relative to the intact group; # *p* < 0.05 relative to the alcoholization group.

The increase in GGT activity from 1.6 ± 0.1 Units/L in the norm (intact group) to 3.4 ± 0.1, 4.4 ± 0.1, and 5.9 ± 0.2 Units/L during alcoholization lasting 3, 6, and 9 months (control), respectively, is an indicator of progressive liver cell destruction (Figure 3). The co-administration of ethanol and C_60_FAS (dose 1 mg/kg) reduced the described indices by 15 ± 1%, 18 ± 1%, and 22 ± 1% during alcoholization of 3, 6, and 9 months, respectively, relative to the control. This index did not significantly change with an increase in the dose of C_60_FAS to 2 mg/kg, and decreasing the dose of C_60_FAS to 0.5 mg/kg reduced its positive effect by 10 ± 1% relative to the group “alcoholization+C_60_ (1 mg/kg)”.

ALP is an enzyme that catalyzes the removal of phosphate groups from organic compounds, such as proteins and nucleotides, and is involved in the transport of phosphorus and calcium in the body. ALP activity in the blood increases as a result of liver diseases, particularly hepatitis caused by alcohol intoxication [22].

ALP activity increased from 8.8 ± 0.5 Units/L in the norm (intact group) to 9.4 ± 0.7, 9.8 ± 0.7, and 10.3 ± 0.7 Units/L during alcoholization lasting 3, 6, and 9 months (control), respectively (Figure 3). The co-administration of ethanol and C_60_FAS (dose 1 mg/kg) decreased ALP activity by 10 ± 1% during the experiment relative to the control. This index did not significantly change with an increase in the dose of C_60_FAS to 2 mg/kg, and reducing the dose of C_60_FAS to 0.5 mg/kg decreased its positive effect by 10 ± 1% relative to the group “alcoholization+C_60_ (1 mg/kg)”.

Transferrin is a blood plasma protein that transports iron ions. The liver is the main source of transferrin production, the main role of which is to deliver iron from absorption centers in the duodenum to the rest of the tissues and digestion of red blood cells by macrophages. The CDT (carbohydrate-deficient transferrin) level is an important biomarker of chronic alcohol intoxication. The advantage of using this index over others is that it is more specific and rarely produces false-positive reactions in liver damage of alcoholic etiology [23].

The CDT level increased from 1.54 ± 0.10% in the norm (intact group) to 1.65 ± 0.1%, 1.75 ± 0.1%, and 1.94 ± 0.1% during alcoholization lasting 3, 6, and 9 months (control), respectively (Figure 3). The co-administration of ethanol and C_60_FAS (dose 1 mg/kg) decreased CDT levels by 10 ± 1% during the experiment relative to the control. This index did not significantly change with an increase in the dose of C_60_FAS to 2 mg/kg, and decreasing the dose of C_60_FAS to 0.5 mg/kg reduced its positive effect by 10 ± 1% relative to the “alcoholization+C_60_ (1 mg/kg)” group.

The effects of C_60_ fullerenes on the in vivo models [19,20] can be explained by their antioxidant properties. To confirm this hypothesis in our case, we assessed the indicators of pro-/antioxidant balance in the blood of the studied rats.

**Figure 4:**
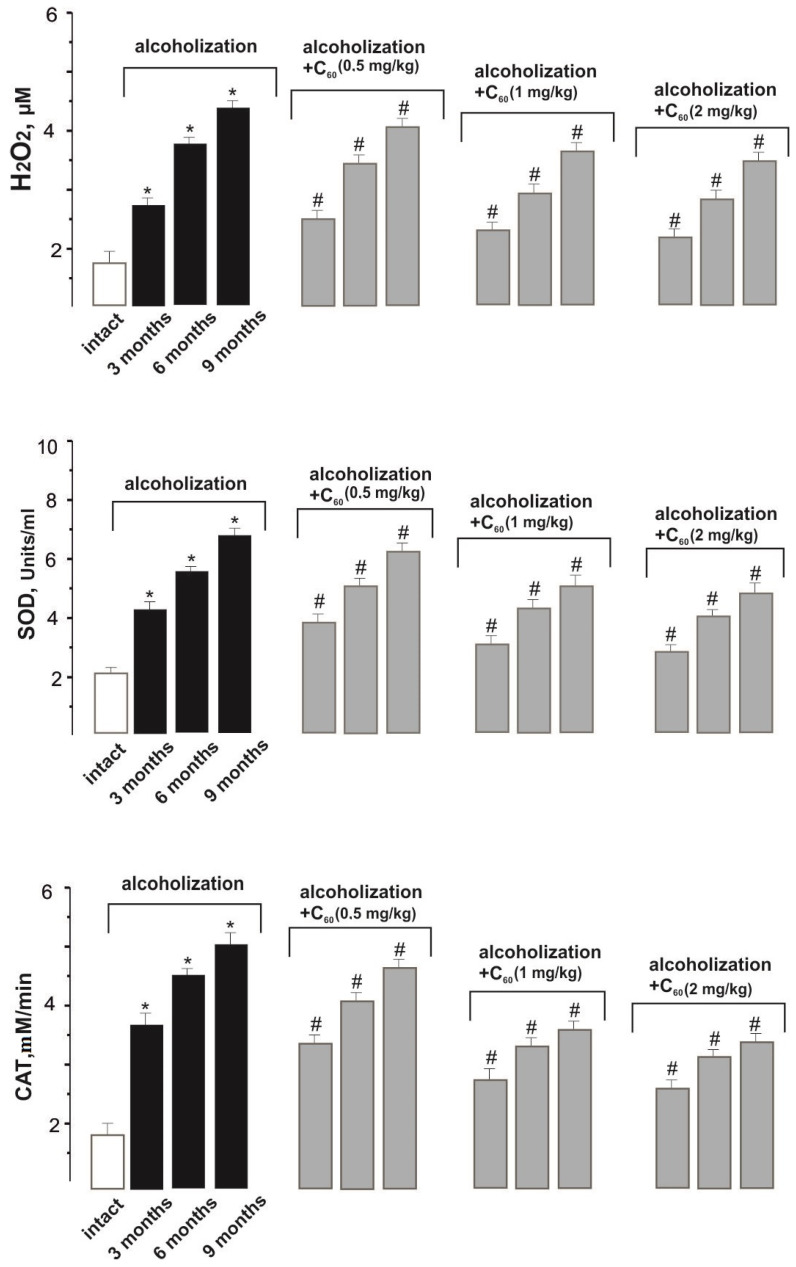

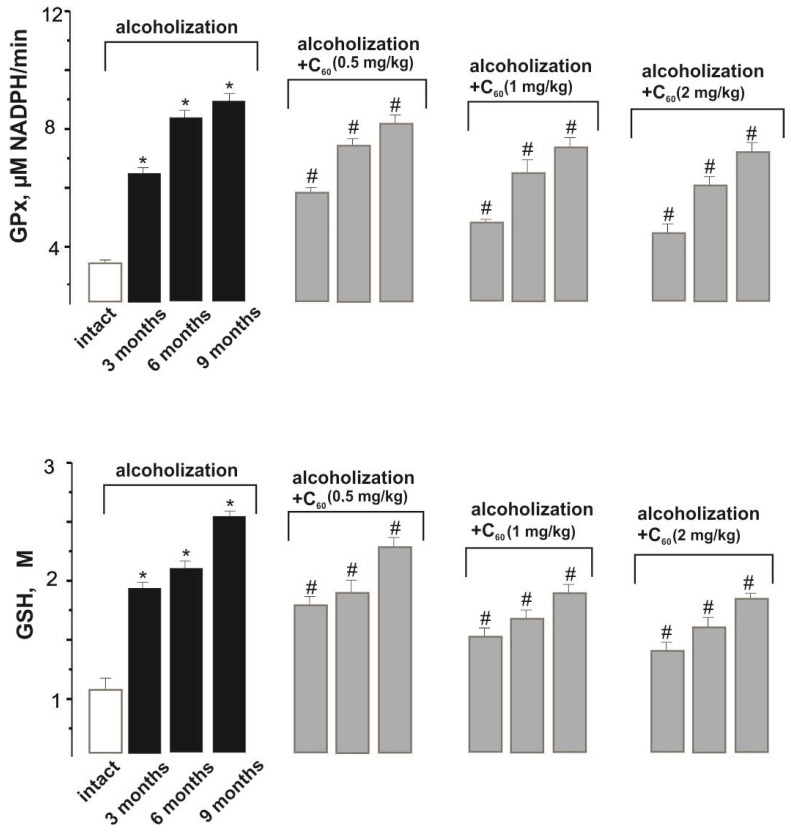
Pro- and antioxidant balance indicators (H_2_O_2_, SOD, CAT, GP_x_, and GSH) in the blood of alcoholized animals: intact—rats receiving 100% drinking water; alcoholization—alcoholized rats (control); alcoholization+C_60_ (0.5, 1, and 2 mg/kg)—rats receiving a mixture of ethanol and C_60_FAS at doses of 0.5, 1, and 2 mg/kg, respectively, throughout alcoholization; 3, 6 and 9 months—alcoholization of 3, 6, and 9 months, respectively. * *p* < 0.05 relative to the intact group; # *p* < 0.05 relative to the alcoholization group.

The data presented in Figure 4 indicate an increased level of LPO and oxidative stress in the blood of rats after prolonged alcohol intoxication. Thus, H_2_O_2_ (hydrogen peroxide) concentration increased by 151 ± 9%, 327 ± 12%, and 414 ± 17% during alcoholization of 3, 6, and 9 months, respectively, compared with the intact group. The co-administration of ethanol and C_60_FAS (dose 1 mg/kg) decreased H_2_O_2_ content by 24 ± 2%, 27 ± 2%, and 20 ± 2% during alcoholization lasting 3, 6, and 9 months, respectively, relative to the control. These data confirm that a water-soluble C_60_ fullerene can penetrate through the cell membranes without signs of their damage [21]. Finally, this index did not significantly change with an increase in the dose of C_60_FAS to 2 mg/kg, and decreasing the dose of C_60_FAS to 0.5 mg/kg reduced its positive effect by 14–16 ± 1% relative to the group “alcoholization+C_60_ (1 mg/kg)”. 

A key role in the mechanisms of regulation of free radical and peroxide processes in the body belongs to such antioxidant defense enzymes as SOD (superoxide dismutase), CAT (catalase), GP_x_ (selenium-dependent glutathione peroxidase), and GSH (reduced glutathione). SOD is the most powerful natural antioxidant and enzyme of the first link of antioxidant defense, which performs the dismutation reaction of superoxide anion radicals and transforms them into less reactive hydrogen peroxide molecules [24].

In the experiment against the background of combined use of ethanol and C_60_FAS (dose 1 mg/kg), a gradual decrease in SOD activity by 25 ± 2%, 29 ± 2%, and 30 ± 2% was found during alcoholization of 3, 6, and 9 months, respectively, relative to the control (Figure 4). This index did not significantly change with an increase in the dose of C_60_FAS to 2 mg/kg, and reducing the dose of C_60_FAS to 0.5 mg/kg decreased its positive effect by 15–18 ± 1% relative to the group “alcoholization+C_60_ (1 mg/kg)”.

CAT activity was decreased by 27 ± 2%, 29 ± 2%, and 30 ± 2% when ethanol and C_60_FAS were co-administered at a dose of 1 mg/kg during alcoholization of 3, 6, and 9 months, respectively, relative to the control (Figure 4). This index did not significantly change with an increase in the dose of C_60_FAS to 2 mg/kg, and decreasing the dose of C_60_FAS to 0.5 mg/kg reduced its positive effect by 14–19 ± 1% relative to the “alcoholization+C_60_ (1 mg/kg)” group.

The cellular mechanisms of the body’s antioxidant defense are also associated with a powerful glutathione link functioning [24]. The glutathione system is one of the active components of antioxidant defense along with antiradical enzymes, which not only prevents the course of free radical reactions but also ensures the effective elimination of the final metabolites of LPO [25].

In the experiment against the background of combined use of ethanol and C_60_FAS (dose 1 mg/kg), a decrease in GP_x_ activity by 24 ± 2%, 25 ± 2%, and 20 ± 2% was found during alcoholization of 3, 6, and 9 months, respectively, relative to the control (Figure 4). This index did not significantly change with an increase in the dose of C_60_FAS to 2 mg/kg, and decreasing the dose of C_60_FAS to 0.5 mg/kg reduced its positive effect by 11–17 ± 1% relative to the group “alcoholization+C_60_ (1 mg/kg)”.

The co-administration of ethanol and C_60_FAS (dose 1 mg/kg) decreased GSH level by 21 ± 2%, 27 ± 2%, and 25 ± 2% during alcoholization lasing 3, 6, and 9 months, respectively, relative to the control (Figure 4). This agrees well with preliminary studies [26], where it was shown that C_60_ fullerenes affect the processes of glutathione synthesis and metabolism in different tissues under various pathologies by modulating the Nrf2/ARE-antioxidant pathway. This index did not significantly change with an increase in the dose of C_60_FAS up to 2 mg/kg, and decreasing the dose of C_60_FAS up to 0.5 mg/kg reduced its positive effect by 11–17 ± 1% relative to the “alcoholization+C_60_ (1 mg/kg)” group.

Summarizing the above biochemical data, we can conclude that 1 mg/kg is the optimal dose of C_60_FAS administration: reducing it by half significantly decreases the therapeutic effect of C_60_ fullerenes, and doubling it does not lead to a significant increase in the observed effects.

### 2.4. Histological Analysis 

**Figure 5:**
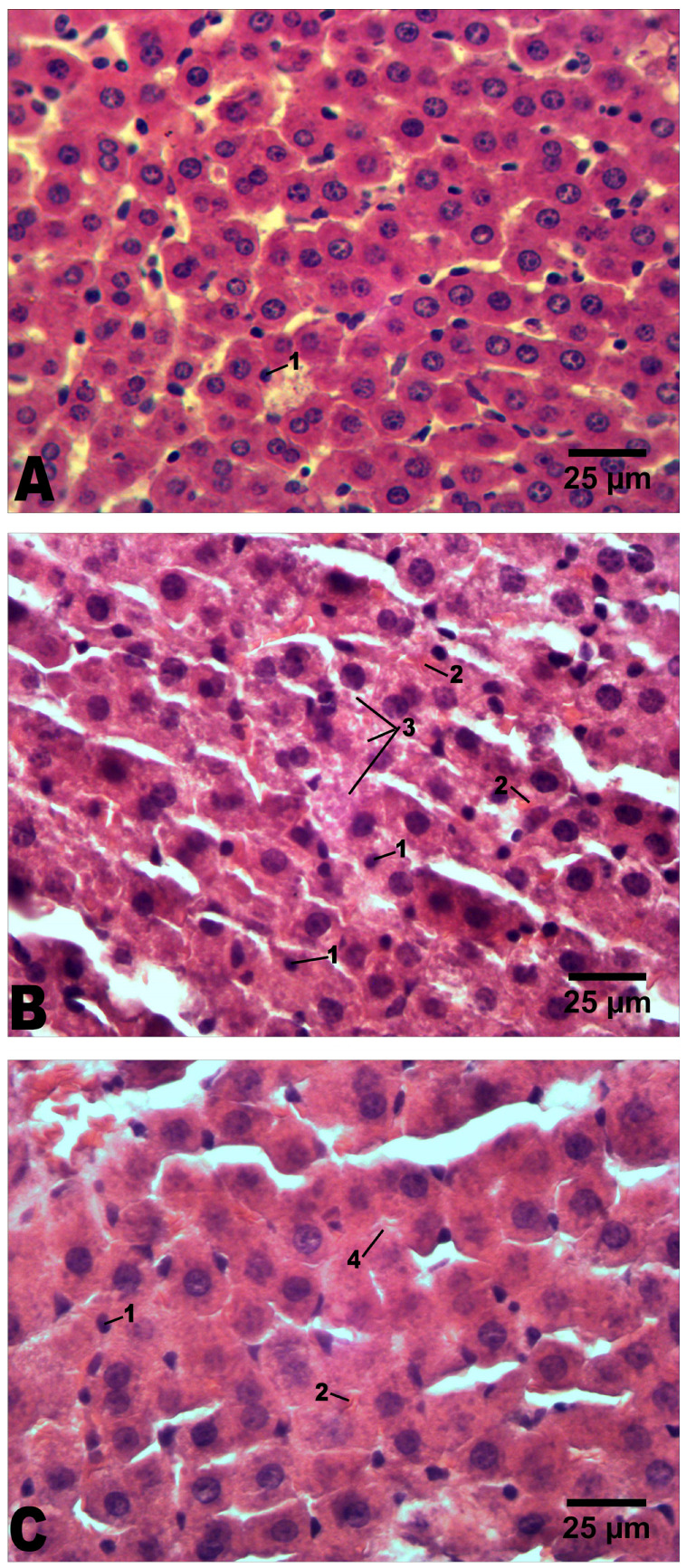
Representative histological images of the liver: (**A**)—intact group; (**B**)—alcoholization group; (**C**)—alcoholization+C_60_ (1 mg/kg) group. H&E (hematoxylin and eosin) staining. Scale bar: 25 μm. 1—pyknotic nuclei; 2—Mallory bodies; 3—small-droplet lipid and balloon dystrophy in alcoholization group; 4—hydropic dystrophy in alcoholization+C_60_ (1 mg/kg) group.

In intact rats, no histopathological features were observed. Only a small number of pyknotic nuclei in hepatocytes were present (Figure 5A). 

In the liver of alcoholized rats, within 9 months (longest experiment duration), there is a strong hydropic dystrophy of hepatocytes with an increase in cell size (as a result of edema or as a result of increased functional load on hepatocytes) (Table 1; Figure 5B). Some of the cell nuclei are pyknotic, others are enlarged and bright. Also, six animals have small-droplet lipid dystrophy, and four animals have strong large-droplet lipid dystrophy and balloon dystrophy of hepatocytes (Figure 5B). Necrotic hepatocytes are found, in great number, in 5 of 10 rats. Some of the blood vessels are dilated, with leukocyte infiltrates around. There is a weak expansion of connective tissue. 

In the liver of alcoholized rats that received C_60_FAS (optimal dose 1 mg/kg), within 9 months, moderately expressed hydropic and weakly expressed small-droplet lipid dystrophy of hepatocytes are observed, and balloon dystrophy is absent (Figure 5C). The sizes of the cells and the volumes of their nuclei are increased (Table 1). Hepatocytes with pyknotic nuclei and necrotic hepatocytes are present but in significantly lower numbers than in alcoholized animals that did not receive C_60_FAS. Occasionally there are dilated blood vessels, with leukocyte infiltrates around. There is a weak expansion of connective tissue (Figure 5C). Therefore, histopathological changes in the liver of rats that received C_60_FAS together with alcohol are clearly smaller. 

The authors [27] showed that more than 90% of alcohol consumed is metabolized through oxidative and non-oxidative pathways with the formation of such reactive compounds as acetate, acetaldehyde, fatty acid ethyl ester, etc. These compounds generate ROSs, which causes an increase in oxidative stress and LPO, thus disrupting the structural integrity of cells and, accordingly, the functions of tissues and organs. In our opinion, a potential mechanism of the C_60_ fullerene effects described above are related to their powerful antioxidant properties [13,14]: by effectively inactivating ROSs, C_60_ fullerenes reduce the number of damaged hepatocytes and thus reduce the severity of alcohol intoxication. The results obtained here are in good agreement with the previous data on the C_60_ fullerene inhibition of liver inflammation and improvement in liver and pancreas state under acute and chronic cholangitis in rats [28]. Moreover, the results of [29,30] indicate an indirect effect of antioxidants on the increase in LPO indicators in alcohol-induced muscle damage. It has been shown that the use of N-acetylcysteine can attenuate the toxic effects of alcohol [31]. A study [32] demonstrated that procysteine use increases mitochondrial glutathione levels and thereby alleviates alcohol-induced oxidative stress.

## 3. Materials and Methods

### 3.1. Preparation and Characterization of C_60_FAS 

A unique method based on the transfer of C_60_ fullerenes from toluene into water with simultaneous ultrasonic treatment (8 Hz, 8 h) was used to prepare the highly stable reproducible C_60_FAS [33]. The resulting dark brown C_60_FAS (maximum concentration 0.15 mg/mL) is stable for 12–18 months in the temperature range of +4–25 °C.

The structural state of C_60_ fullerene was studied using AFM after deposition of its layer from a drop of C_60_FAS on a mica substrate and evaporation of water. AFM measurement was performed using the “Solver Pro M” (NT-MDT) system in the amplitude modulation tapping mode using probes “RTESPA-150” (Bruker, Preston, VIC, Australia, ~150 kHz, 5–10 N/m, R_tip_ < 12 nm). The free oscillations’ amplitude of the cantilever was set to 50 nm. No image processing techniques other than surface tilt subtraction were used.

### 3.2. In Vivo Experiments 

The experiments were carried out on male Wistar rats aged 1 to 10 months (at the end of the experiment). The initial weight of the animals was 170 ± 10 g. 

The study protocol was approved by the Bioethics Committee of the ESC “Institute of Biology and Medicine” of Taras Shevchenko National University of Kyiv (protocol No. 2 dated 2 September 2022) in accordance with the rules of the European Convention for the Protection of Vertebrate Animals Used for Experimental and Other Scientific Purposes and the norms of biomedical ethics in accordance with the Law of Ukraine No. 3447—IV of 21 February 2006, Kyiv, “On the Protection of Animals from Cruelty”, as well as the European Union Directive (2010/63/EU) “On the protection of animals used for scientific purposes”.

During the experiment, we used the following groups of animals:

- The intact group of rats (n = 10) received 100% drinking water;
- Rats in the “alcoholization” group (control; n = 30; 10 animals in each group) were randomly selected. Each rat was placed in a separate cage to receive 40% ethanol in drinking water [34], which meant that the animals did not have access to 100% water until they had consumed the dosed portion of ethanol [18]. The amount of ethanol consumed was calculated with respect to 0.5% of the animal’s body weight. Recalculation of the ethanol dose was performed every 24 h throughout the experiment [35]. The duration of alcoholization was 3, 6, and 9 months. The target value of 40% ethanol in drinking water was chosen because it reflects the blood alcohol concentrations reported in chronic alcoholics [32];
- Rats of the “alcoholization+C_60_” groups (n = 90; 10 animals in each group) together with ethanol were orally administered C_60_FAS at daily doses of 0.5, 1, and 2 mg/kg of rat weight. Recalculation of the C_60_FAS dose was performed every 24 h throughout the experiment. Control of the amount of C_60_FAS ingested was performed by denying the animals access to drinking 100% water until they fully consumed the applied drug.

Note that the tested doses of C_60_FAS are significantly lower than the LD_50_ value, which was 600 mg/kg body weight when administered orally to rats [36] and 721 mg/kg when administered intraperitoneally to mice [17]. It has been established that after intravenous administration to mice, C_60_ fullerenes accumulate predominantly in the blood, spleen, stomach, and liver and are excreted from the body within 72 h, mainly with urine [37].

Finally, it should also be emphasized that in our recent research [38], various regimens of C_60_FAS administration (1 mg/kg) were studied during rat alcoholization lasting 3, 6, and 9 months, namely, 1 h before drinking alcohol, together with alcohol, and 1 h after drinking alcohol. Based on the assessment of the biochemical indices of blood and tissue of the *muscle gastrocnemius* chronically alcoholized rats, the most optimal regimen for administering C_60_FAS was determined, namely, together with alcohol, the positive effects of which exceeded other regimens for the administration of this pharmacological agent.

Animals in the study groups were euthanized (carbon dioxide-induced hypoxia) on the last day of the experiment, and all efforts were made to minimize suffering. During the study, animals were observed daily for visual clinical signs according to the FELASA recommendations [39].

### 3.3. Biochemical Analysis 

The activities of the enzymes ALT, AST, GGT, and ALP, the level of CDT, and the values of pro-antioxidant balance indicators (H_2_O_2_ and GSH contents, CAT, SOD, and GP_x_ activities) in the blood of rats as markers of liver dysfunction were determined using clinical diagnostic equipment—biochemical analyzers RNL-200 (The Netherlands) and ABX Micros ESV60 (France).

The BAC value in rats was measured at the end of experiment using an AM1 alcohol analyzer (Analox Instruments Limited, Stourbridge, UK).

### 3.4. Histological Analysis 

The liver samples were separated and fixed in 10% formalin. They were then embedded in paraffin, cut into 5 μm thick sections, and stained with H&E [40]. Digital micrographs of stained sections were taken at a magnification of ×400 by using a computer-assisted image analyzing system (Olympus BX41 microscope and Olympus C-5050 Zoom digital camera). The histopathological profiles of the liver were determined by light microscopy observation. Intensity of each histopathological feature was evaluated as follows: “–”—not observed; “+”—slight; “++”—moderate; “+++”—strong. Diameter of hepatocytes’ nuclei and cross-sectional area of hepatocytes were measured using ImageJ software [41]. Volume of hepatocytes’ nuclei was calculated using formula $V=\frac{\pi }{6}D^{2}$, where *D* is the diameter of hepatocytes’ nuclei.

### 3.5. Statistics 

The experimental results were processed by methods of variation statistics using the Statistica 13.3 program. At least three repeats were performed for each measurement. A *t*-test (for one experimental group) or one-way ANOVA (for three experimental groups) followed by a post hoc Dunnett multiple comparison test to compare all data vs. control were used. A factorial ANOVA test was also used depending on such factors of variation as dose and treatment period. Assumptions that the data followed a normal distribution and had identical standard deviations were tested using the Shapiro–Wilk and Bartlett methods, respectively. The differences among experimental groups were considered statistically significant at *p* < 0.05. 

## 4. Conclusions

Thus, in all tests performed after chronic alcoholization during 3, 6, and 9 months, when alcohol and C_60_FAS at the optimal dose of 1 mg/kg were co-administered, there was a positive change in the studied biochemical parameters of rat blood, as markers of liver damage, up to 29 ± 2% relative to the control. Ultimately, the above biochemical results were confirmed by histopathological data. It can be concluded that the use of C_60_FAS contributes to the reduction in oxidative processes in tissues by maintaining a balance between pro-oxidants and the body’s antioxidant defense system. This prevents the negative impact of ROSs on cellular and subcellular structures during prolonged alcohol intoxication. On this basis, it can be assumed that C_60_ fullerenes, as powerful nanoantioxidants, are able to correct pathological conditions of the liver arising in chronic alcohol intoxication, which requires further clinical trials. 

## Figures and Tables

**Table 1:** Histopathological features of the liver in rats after alcohol and C_60_FAS consumption within 9 months.

	Groups	Intact	Alcoholization	Alcoholization+C_60_ (1 mg/kg)
Histopathological Features				
Volume of hepatocyte’s nucleus, µm^3^		138 ± 8	248 ± 11 *	284 ± 19 ^#^
Cross-sectional area of hepatocyte, µm^2^		205 ± 11	273 ± 20 *	244 ± 11 ^#^
Pyknotic nuclei in hepatocytes		+	++	+
Mallory bodies		–	+++	++
Lipid dystrophy of hepatocytes		–	+++	+
Hydropic dystrophy of hepatocytes		–	+++	++
Balloon dystrophy of hepatocytes		–	++	–
Necrotic hepatocytes		–	++	+
Blood vessel dilatation		–	++	+
Inflammatory cell infiltration		–	++	++
Expansion of connective tissue (fibrosis)		–	+	+

Trait intensity: “–”—not observed; “+”—weak; “++”—moderate; “+++”—strong. * *p* < 0.05 compared with the intact group; ^#^ *p* < 0.05 compared with the alcoholization group.

## Data Availability

Dataset available upon request from the authors.
